# The impact of large-scale deployment of *Wolbachia* mosquitoes on dengue and other *Aedes*-borne diseases in Rio de Janeiro and Niterói, Brazil: study protocol for a controlled interrupted time series analysis using routine disease surveillance data

**DOI:** 10.12688/f1000research.19859.2

**Published:** 2020-06-11

**Authors:** Betina Durovni, Valeria Saraceni, Ana Eppinghaus, Thais I.S. Riback, Luciano A. Moreira, Nicholas P. Jewell, Suzanne M. Dufault, Scott L. O'Neill, Cameron P. Simmons, Stephanie K. Tanamas, Katherine L. Anders

**Affiliations:** 1 Centre for Strategic Studies, Fiocruz, Rio de Janeiro, Brazil; 2 World Mosquito Program, Fiocruz, Rio de Janeiro, Brazil; 3 City Health Secretariat, Rio de Janeiro, Brazil; 4 City Health Secretariat, Niteroi, Brazil; 5 Instituto Rene Rachou, Fiocruz, Belo Horizonte, Brazil; 6 Division of Epidemiology and Biostatistics, School of Public Health, University of California, Berkeley, Berkeley, CA, USA; 7 Centre for Statistical Methodology, London School of Hygiene & Tropical Medicine, London, UK; 8 World Mosquito Program, Institute of Vector Borne Disease, Monash University, Melbourne, VIC, Australia

**Keywords:** Wolbachia, dengue, chikungunya, Zika, vector-borne disease, disease surveillance, controlled interrupted time series, Brazil

## Abstract

**Background: **Rio de Janeiro and Niterói are neighbouring cities in southeastern Brazil which experience large dengue epidemics every 2 to 5 years, with >100,000 cases notified in epidemic years. Costs of vector control and direct and indirect costs due to the *Aedes*-borne diseases dengue, chikungunya and Zika were estimated to total $650 million USD in 2016, but traditional vector control strategies have not been effective in preventing mosquito-borne disease outbreaks. The *Wolbachia* method is a novel and self-sustaining approach for the biological control of *Aedes*-borne diseases, in which the transmission potential of *Aedes aegypti* mosquitoes is reduced by stably transfecting them with the *Wolbachia* bacterium ( *w*Mel strain). This paper describes a study protocol for evaluating the effect of large-scale non-randomised releases of *Wolbachia­*-infected mosquitoes on the incidence of dengue, Zika and chikungunya in the two cities of Niterói and Rio de Janeiro. This follows a lead-in period since 2014 involving intensive community engagement, regulatory and public approval, entomological surveys, and small-scale pilot releases.

**Method:** The *Wolbachia* releases during 2017-2019 covered a combined area of 170 km ^2^ with a resident population of 1.2 million, across Niterói and Rio de Janeiro. Untreated areas with comparable historical dengue profiles and demographic characteristics have been identified *a priori* as comparative control areas in each city. The proposed pragmatic epidemiological approach combines a controlled interrupted time series analysis of routinely notified suspected and laboratory-confirmed dengue and chikungunya cases, together with monitoring of *Aedes*-borne disease activity utilising outbreak signals routinely used in public health disease surveillance.

**Discussion:** If the current project is successful, this model for control of mosquito-borne disease through *Wolbachia* releases can be expanded nationally and regionally.

## Abbreviations

BG trap: BG-Sentinel trap; IBGE: Instituto Brasileiro de Geografia e Estatística (Brazilian Institute of Geography and Statistics); ITS: interrupted time series; MoH: Ministry of Health; PAHO: Pan American Health Organisation; PCR: polymerase chain reaction; qPCR: quantitative polymerase chain reaction; RCT: randomised controlled trial; SINAN: Sistema De Informação De Agravos De Notificação (Brazilian National Notifiable Diseases Information System); WHO: World Health Organisation

## Background

The global incidence of dengue has increased dramatically in recent decades. Although cases are underreported, it is estimated that 390 million dengue virus infections occur every year, and of these 96 million have clinical manifestations of dengue or severe dengue. Globally, 3.9 billion people in 128 countries are at risk of infection ^1^. The primary vector of dengue is the *Aedes aegypti* mosquito, which is also capable of transmitting other arboviruses (i.e. mosquito-borne viruses) including chikungunya, Zika, yellow fever and Mayaro ^2^.

The first reported dengue outbreak in Brazil was in 1845, with subsequent outbreaks in 1880–1912 and 1916–1923 ^2^. As a result of a coordinated effort from the Pan American Health Organization (PAHO) and the World Health Organization (WHO) to eradicate *Ae. aegypti*, Brazil was considered free of the mosquito in 1955, but the vector was reintroduced into the country two decades later ^3^. In 1986, dengue virus serotype 1 (DENV1) was introduced to Rio de Janeiro and an estimated 1 million people were infected ^3, 4^. Since then, dengue has become a major public health problem. From 1986 to 1993, outbreaks occurred approximately every 2–5 years, and from 1993 dengue became endemic with seasonal peaks in cases during the rainy season (December to May), but with ongoing transmission throughout the year ^2, 3^. Between 2000 and 2007, more than 3 million dengue cases were reported (caused by DENV serotypes 1, 2 and 3) and in 2010, DENV4 re-emerged after 28 years of absence ^2^.

The recent introduction of Zika and chikungunya in dengue hyperendemic areas of Brazil has aggravated the situation. The overlapping clinical features, absence of serological assays for the Zika virus that can reliably distinguish between acute disease and past exposure, and the association of pregnancy-associated Zika virus infection with microcephaly and other neurologic complications represents a great challenge for public health that will require new strategies and innovations ^5, 6^.

Between 2016 and 2017, 762 deaths were attributed to severe dengue in Brazil ^7^. Additionally, in 2017, 127 deaths were confirmed to be caused by chikungunya. Yellow fever has spread from the North of Brazil to the Southeast over the last years, affecting humans and non-human primates. From July 2017 to April 2018, 1,266 cases of yellow fever including 415 deaths were confirmed in Brazil, with 223 cases and 73 deaths occurring in Rio de Janeiro State ^8^. No autochthonous cases were reported in Rio de Janeiro city or Niterói, although some residents from those cities acquired yellow fever while traveling to other places in Brazil. A mass vaccination campaign against yellow fever began in 2016.

The costs of vector control, direct medical costs, and indirect costs related to dengue, Zika and chikungunya in Brazil were estimated to be 2.3 billion Brazilian reais ($650 million USD) in 2016 ^9^. In the absence of an effective vaccine for these arboviruses, disease prevention depends on vector control. Vector control guidelines in Brazil ^10^ are focused on elimination or larvicide treatment of mosquito breeding sites and the control of adult mosquito populations with insecticides sprayed as ultra-low volume. The limited potential of these traditional vector control strategies to achieve large-scale and sustained reductions in dengue incidence is evidenced by the continuing public health burden of dengue throughout endemic areas where these measures are routinely employed, and the lack of robust efficacy data from well-designed trials to inform their optimal implementation ^11^.

The *Wolbachia* method ( www.worldmosquito.org) is a novel, natural, and self-sustaining approach to reduce arboviral diseases transmitted by *Ae. aegypti* mosquitoes. The symbiotic *Wolbachia* bacterium is found naturally in over 60% of insect species, but not in *Ae. aegypti*, and is passed from one generation to the next through the insect’s eggs. Stable transinfection of *Wolbachia* into a local *Ae. aegypti* colony in the laboratory produces a lineage of *Wolbachia-*carrying mosquitoes which, upon release over several weeks, can achieve dissemination of *Wolbachia* into the local *Ae. aegypti* population through the processes of maternal inheritance and cytoplasmic incompatibility that give *Wolbachia*-carrying mosquitoes a reproductive advantage. The DENV-transmitting potential of mosquitoes stably transfected with *Wolbachia* *pipientis* ( *w*Mel strain) is reduced by 66–75% ^12, 13^, a phenotype which has been shown to persist in field mosquito populations up to five years after the end of releases. Mathematical modelling of this reduced transmissibility predicts a substantial and sustained reduction in dengue incidence in human populations where *Wolbachia* is established ^13^. Laboratory data indicate a similar reduction in the competence of *Wolbachia-*carrying *Ae. aegypti* for transmitting other viruses including Zika, chikungunya, yellow fever and Mayaro ^14– 17^.

With releases now conducted in eight countries over the past eight years, the World Mosquito Program has demonstrated that *Wolbachia* can be successfully established and maintained in both small-scale and large-scale urban settings in multiple ecological environments ^18– 22^.

A core objective of these releases has been to ensure strong community acceptance and government support for the approach, achieved through embedding community and stakeholder engagement within the project activities in each site. Observational evidence of the impact of *Wolbachia* releases on arboviral disease in pilot sites has been encouraging, with no evidence of local dengue transmission where *Wolbachia* has established at high levels. Following city-wide deployment in Townsville, Australia, there has been no confirmed local dengue transmission in *Wolbachia*-treated areas for four seasons since completion of releases, despite local transmission every year for the prior 13 years and ongoing importation of DENV infection in travelers ^21^. A cluster randomized controlled trial (RCT) to generate a robust and quantitative estimate of the impact of *Wolbachia* on dengue incidence commenced in Yogyakarta, Indonesia in 2017, with reporting of results expected in 2021 ^23^.

In Brazil, planned scale up of *Wolbachia* deployments from demonstration projects in Rio de Janeiro and Niterói to large-scale releases was accelerated by the declaration of Zika as a public health emergency by the WHO in early 2016, and the recommendation by WHO’s Vector Control Advisory Group in March 2016 that the *Wolbachia* method be evaluated in rigorously monitored pilot deployments under operational conditions, to build evidence of epidemiological effectiveness against *Aedes*-borne viruses ^24^. Given the imperative from stakeholders and funders to scale up deployment within a relative short time frame, and to retain sufficient flexibility to optimize methods for large-scale deployment in the varied micro-environments within Niterói and Rio de Janeiro, an RCT or other carefully controlled deployment was not considered feasible. Instead releases under operational conditions, and with pragmatic evaluation of disease impact using data routinely collected for public health purposes, was favoured. The primary reason that randomised deployment of the *Wolbachia* intervention was not undertaken (either by random allocation of treated and untreated areas, or by randomising the sequence of staged deployments) was the intensive community engagement effort required in release areas in advance of deployments, as well as the gains in logistical efficiency and entomological outcomes from staging deployments across large contiguous areas. The potential benefits of randomised allocation of deployments in reducing bias from known and unmeasured confounders would require the intervention (and control) areas to be divided into a large number of units for randomisation. This fragmentation of the deployment areas would have made the community engagement activities and deployment logistics infeasible. Having small, fragmented release areas interspersed with untreated areas would also have greatly increased the potential for contamination between treatment arms from both mosquito movement and human movement, thereby diluting the observable intervention effect.

Here we describe a protocol for evaluating the effect of large-scale non-randomized *Wolbachia* releases on the incidence of dengue, chikungunya and Zika in the cities of Niterói and Rio de Janeiro. The proposed strategy employs a controlled interrupted time series analysis of routinely notified suspected and laboratory-confirmed cases of dengue, chikungunya and Zika, together with monitoring of disease activity with outbreak signals routinely used in public health disease surveillance. This methodology allows measurement of the impact of the intervention at the population level over time, accounting for the seasonal trends and inter-annual fluctuations often observed in dengue and other mosquito-borne disease incidence.

## Methods

### Study design

The aim of this epidemiological study is to test the hypothesis that the establishment of *Wolbachia* in local *Ae. aegypti* populations in Rio de Janeiro and Niterói leads to a reduction in the burden of *Aedes*-borne disease.

The impact of *Wolbachia* deployment on disease incidence will be evaluated using routine notifiable disease surveillance data to describe associations between temporal and spatial trends in dengue, chikungunya and Zika and the deployment of *Wolbachia* across Niterói and Rio de Janeiro, with two objectives:

1. Estimate the reduction in dengue, chikungunya and Zika in the aggregate treated areas of Niterói and Rio de Janeiro compared to an untreated control area, and in each treated zone compared to the untreated control area, each year for five years after *Wolbachia* establishment.
2. Quantify the occurrence of dengue outbreak signals in *Wolbachia* treated areas compared to untreated areas in Niterói and Rio de Janeiro, for five years after *Wolbachia* establishment, using outbreak indicators employed routinely for public health monitoring of dengue activity: i) control diagrams comparing the weekly dengue incidence (five-week moving average) against the five-year historical average in that same area; and ii) outbreak incidence threshold of 300/100,000 population in any month.

### Study setting and population

Rio de Janeiro and Niterói cities are located in the State of Rio de Janeiro, Brazil. Niterói has an area of 134 km ^2^ and a population of 484,918 in 2010. Rio de Janeiro is the second largest city in Brazil with 6,320,446 inhabitants in 2010 and an area of 1,200 km ^2^. The two cities sit on opposite sides of the Guanabara Bay and are linked by a long bridge, which transports a large commuter population between Niterói and Rio de Janeiro.

**Figure 1.**
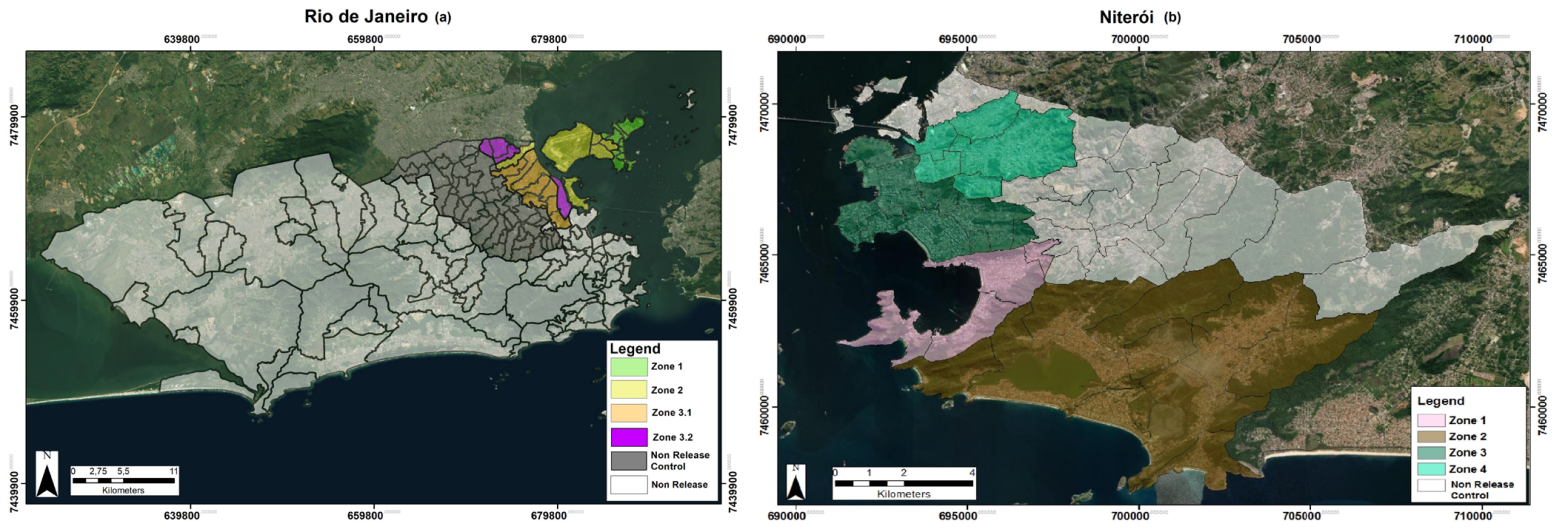
Map of ( **a**) Rio de Janeiro and ( **b**) Niterói *Wolbachia-*treated and untreated areas (produced in ArcMap version 10.5, ESRI, CA).

The cities are divided into health districts, for the purpose of planning and delivering care - ten in Rio de Janeiro and seven in Niterói. In Rio de Janeiro, *Wolbachia* deployments will be conducted in one of the administrative areas of the city ( Figure 1; produced in ArcMap version 10.5, ESRI, CA), in an area of approximately 90 km ^2^ and with 886,551 inhabitants, 39% of whom live in slums. The total release area in Niterói is approximately 83 km ^2^ covering a population of 373,117 ( Table 1). For the purposes of *Wolbachia* deployment, the Rio de Janeiro and Niterói intervention areas are each divided into 4 release zones, respectively, which are aligned with neighbourhood administrative boundaries.

**Table 1.** Demographic characteristics of *Wolbachia* treated and untreated areas, Niterói and Rio de Janeiro (source: 2010 Brazil population census). Neighbourhoods Population Area (Km ^2^) Population density

	Neighbourhoods	Population	Area (Km ^2^)	Population density
Niterói				
Release Zone 1	4	23,747	9.2	2,581
Release Zone 2	11	68,695	50.6	1,357
Release Zone 3	13	178,891	12.6	14,197
Release Zone 4	5	101,784	10.8	9,424
Non-release control area	19	111,801	51.2	2,183
Total	52	484,918	134.4	3,608
Rio de Janeiro				
Release Zone 1	10	107,130	11.8	9,078
Release Zone 2	6	150,646	33.5	4,496
Release Zone 3.1	8	408,036	28.2	14,469
Release Zone 3.2	4	220,739	12.0	18,394
Non-release control area	51	1,512,608	117.3	12,895
Rest of city (no releases)	79	3,921,287	996.5	3,935
Total	158	6,320,446	1,199.3	5,270

**Figure 2.**
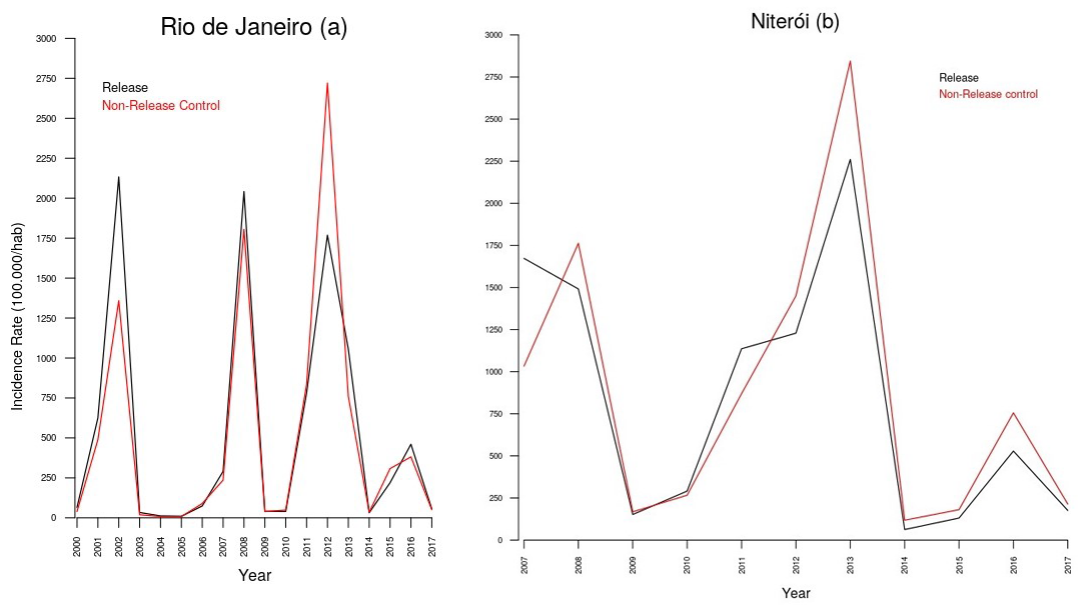
Dengue incidence in *Wolbachia-*treated vs untreated areas of ( **a**) Rio de Janeiro (2000–2017) and ( **b**) Niterói (2007–2017) (source: Brazilian National Disease Surveillance System (SINAN)).

**Figure 3.**
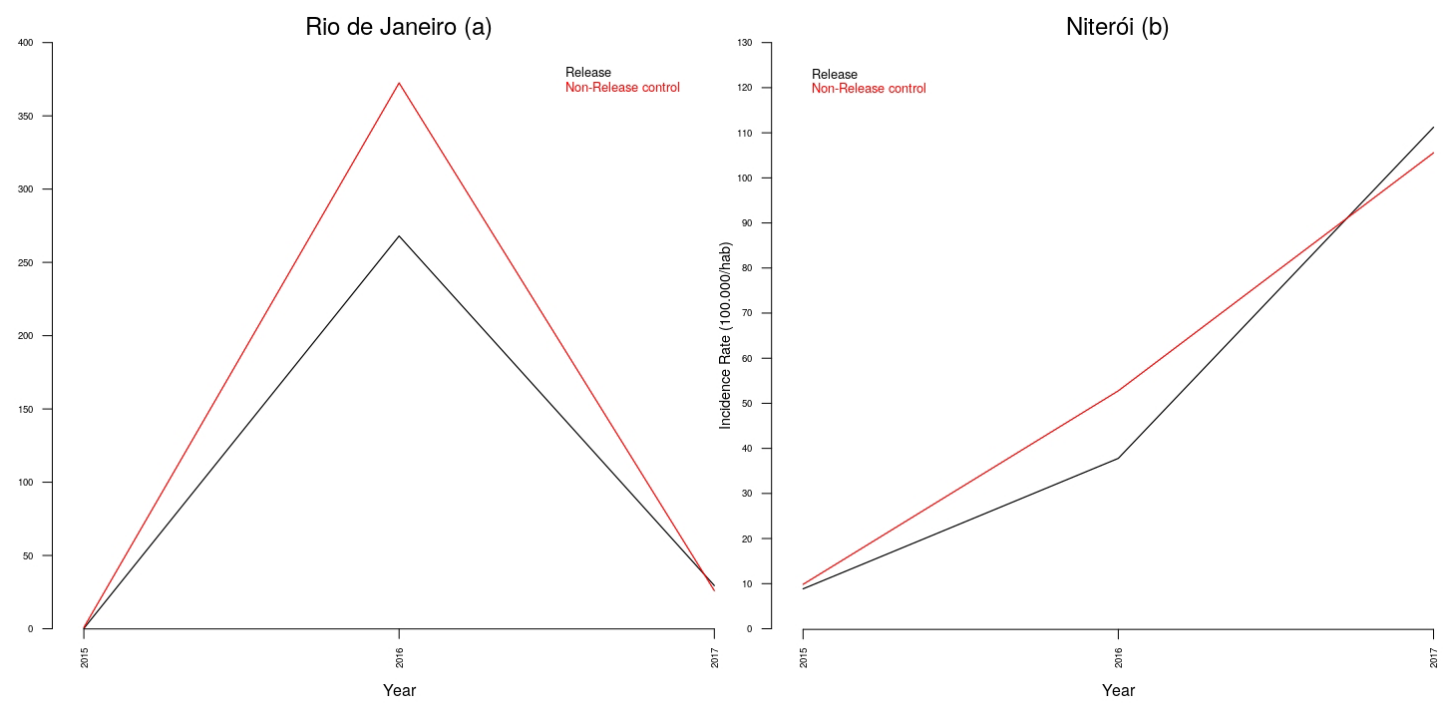
Chikungunya incidence in *Wolbachia*-treated vs untreated areas of ( **a**) Rio de Janeiro (2015–2017) and ( **b**) Niterói (2015–2017) (source: Brazilian National Disease Surveillance System (SINAN)).

In Rio de Janeiro, two administrative areas adjacent to the release area have been designated *a priori* as a comparative control zone ( Figure 1), based on synchronous historical dengue and chikungunya time series ( Figure 2 and Figure 3). In Niterói, the remaining untreated area of the city has been designated as the comparative control zone ( Figure 1).

### Wolbachia release and monitoring

Staged *w*Mel *Wolbachia*-mosquito deployments will be implemented in Niterói and Rio de Janeiro, in order to achieve *Wolbachia* establishment across the two cities. Pilot releases commenced in late 2015, and city-wide deployments are ongoing through to the end of 2019. *Wolbachia*-containing adult mosquitoes will be released at one location per 50 x 50 meter grid square for a minimum of 16 consecutive weeks in each release zone. In areas where *Wolbachia* frequency remains low after 16 weeks of releases, or where particularly high wild type *Ae. aegypti* populations are observed, *Wolbachia*-containing mosquito eggs will also be released to complement adult releases with the aim of accelerating *Wolbachia* establishment. Monitoring of *Wolbachia* frequency will be done using BG-Sentinel mosquito traps (BioGents, Germany), distributed throughout the release area at a density of 16 traps per km ^2^. Traps will be serviced weekly and all collected mosquitoes identified morphologically by microscopy. From eight weeks after the start of releases, a maximum of 10 *Ae. aegypti* (male and female) per trap will be tested individually for *Wolbachia* using quantitative polymerase chain reaction (qPCR). *Wolbachia* screening will be performed biweekly during releases and until establishment, then every 1–3 months thereafter. All surplus *Ae. aegypti* will be biobanked. Mosquito collection and screening results will be stored in a custom designed web-based data repository. The *Wolbachia* prevalence in screened *Ae. aegypti* will be reported aggregated to each release zone, calculated as the total number of *Ae. aegypti* mosquitoes that tested positive for *Wolbachia* aggregated across all BG traps in the zone, divided by the total number of *Ae. aegypti* that were screened in that zone.

### Epidemiological data sources

The two proposed strategies for the evaluation of the impact of large-scale *Wolbachia* deployments on arboviral disease incidence in Niterói and Rio de Janeiro make use of existing data on dengue, chikungunya and Zika case notifications to the Brazilian national disease surveillance system (SINAN). Dengue surveillance has been in place since its re-emergence in 1986 and data is available from the SINAN system since 2000. Zika and chikungunya became notifiable diseases in 2015.

Suspected cases of dengue and other arboviral diseases are required to be reported to the city health department ^25^, according to a case definition of fever plus two other symptoms including malaise, headache, myalgia, nausea, vomiting, cutaneous rash, and arthralgia. Dengue case notifications include an indication of disease severity (dengue, dengue with alarm signals, severe dengue, fatal dengue). A variable proportion of notified suspected cases are tested by IgM serology or PCR, following the Brazilian guidelines ^26^ and the timing between onset of symptoms and blood collection. The number of cases in a given period of time may limit the availability of tests, and PCR testing is routinely performed only for severe and fatal cases, pregnant women and young children ^26^. In 2016, 4.8% of notified dengue cases in Rio de Janeiro and 11.5% in Niterói were supported by a positive IgM serology result, and only 0.2% of notified cases in Rio and 0.03% in Niterói had a positive PCR result. The ability to confirm dengue cases by serology is impaired since the Zika outbreak due to serological cross-reactivity between the dengue and Zika viruses. As PCR testing is performed only in certain patient populations, the proportion positive is unlikely to be generalisable to all notified cases. Therefore, for the purpose of our analyses we will use all notified dengue cases (suspected and laboratory-confirmed) as the primary endpoint.

In the absence of a reliable serological test that does not cross-react with dengue, Zika lab diagnosis is done solely on the basis of molecular detection (real-time PCR) up to the first 5 days in serum and 15 days in urine. This has severely limited the ability to confirm Zika virus infection among notified cases (3.5% in 2016, 19.3% in 2017).

Chikungunya diagnoses can be confirmed either through PCR or serology, as it does not cross-react with Zika or dengue. The proportion of notified cases with supportive laboratory findings is higher than for dengue: 31.9% and 18.2% in Rio and Niterói, respectively, in 2016.

Anonymized disaggregate (line-listed) data on notified suspected and laboratory-confirmed dengue, chikungunya and Zika cases will be extracted from the SINAN system for the historical pre-intervention period (2000–2016 for dengue, and 2015–2016 for chikungunya and Zika) and the prospective post-intervention period (2017–2023). The dataset will include age, sex, neighbourhood of primary residence, date of illness onset, date of notification, reporting health clinic, disease severity, hospitalisation, death, and where available, geocoordinates of primary residence, type of diagnostic test performed, diagnostic test result, and final diagnostic classification. Population data from the Brazilian census (IBGE) and population by neighbourhood of residence obtained from the cities of Rio and Niterói will be used to estimate the population in each release zone. The incidence rate (number of new dengue, Zika or chikungunya cases divided by the population at risk) will be expressed per 100,000 inhabitants.

### Controlled interrupted time series analysis

Interrupted time series (ITS) analysis is a valuable study design for evaluating the effectiveness of a population-level health intervention that is implemented at a clearly defined point in time ^27^. It uses a set of historical observations of an outcome of interest (in this context monthly dengue case notifications) to establish an underlying trend, which is assumed to be ‘interrupted’ by the introduction of an intervention (in this case *Wolbachia* releases). Comparison of the trend in monthly case notifications in the post-intervention period with the hypothetical scenario of no intervention (the ‘counterfactual’, inferred from the historical time series and the untreated control area), provides an estimate of the intervention effect. Segmented regression will be used to estimate the effect of *Wolbachia* releases on monthly case counts of dengue and chikungunya, using an appropriately defined model: e.g. negative binomial regression for autocorrelated count data with population offset, adjusted for temporal effects using indicator variables or flexible cubic splines, and assuming a step change post-intervention. The *Wolbachia* intervention effect will be estimated from the interaction between a binary ‘area’ variable (intervention vs control area) and a binary ‘period’ variable (post *-*intervention ** vs ** pre *-*intervention). The post-intervention period will be defined for the control area beginning at the same time as the post-intervention period in the comparative release zone. This allows explicitly for a level change in the outcome (dengue case incidence) in both intervention and control areas in the post-intervention period, for example in a scenario where other secular events coincident with the *Wolbachia* deployments may have influenced dengue incidence in both intervention and control areas independent of *Wolbachia*. An additional analysis will consider *Wolbachia* frequency as a continuous covariate or categorised into quintiles of exposure reflecting the measured *Wolbachia* prevalence in the local mosquito population. The outcome distribution is assumed to be negative binomial to allow for overdispersion. Robust standard errors will be used to account for autocorrelation and heteroskedasticity. If an excess of zero counts is apparent, the proposed negative binomial model will be nested within a zero-inflated negative binomial model of the same structure. A likelihood ratio test can be performed to test model fit.

Separate analyses will be performed for each release zone compared with its pre-defined control area, and for the aggregate release areas in each city compared with the control area for each city. The availability of comparative control areas – well-matched to the release area in demographic characteristics and historical arboviral disease incidence ( Figure 2 and Figure 3) – permits a robust, controlled analysis in which the confounding effects of seasonality and inter-annual variability can be adjusted for. The intervention effect will be estimated after 12 months of post-release observations have accumulated, and each 12 months thereafter for five years using cumulative post-release observations, for each release zone individually and in aggregate for each city.

Power was estimated for the ITS analysis using 1000 simulated datasets drawn from a negative binomial distribution fitted to a ten-year time series (2007–2016) prior to *Wolbachia* deployment, of monthly dengue case notifications from release and control zones in Niterói and Rio de Janeiro. The simulated time series of dengue case numbers in the control zones as well as the pre- *Wolbachia* release dengue case numbers in the treated zones were drawn directly from this model-generated distribution. Post- *Wolbachia* release dengue case numbers in the treated zones were drawn from the same model-generated distribution, modified by an additional parameter for an intervention effect of Relative Risks = 0.6, 0.5, 0.4, 0.3. For each of these four ‘true’ effect sizes and a null effect (RR = 1), applied to each of the 1000 simulated time series, the ‘observed’ effect size was calculated from a negative binomial regression model of monthly case counts in the treated and untreated zones, as described above. Post-intervention time periods of 1, 2 or 3 years were simulated, with the pre-intervention period fixed at 7 years. The estimated power to detect a given effect size was determined as the proportion of the 1000 simulated scenarios in which a significant intervention effect (p<0.05) was observed. These simulations indicate 80% power to detect a reduction in dengue incidence of 50% or greater after three years of post-intervention observations, and a reduction of 60% or greater after two years.

The primary endpoint for the ITS analysis will be dengue cases notified to the disease surveillance system. The secondary endpoints will be: a) the count of severe dengue cases reported to the surveillance system, b) the count of fatal dengue cases reported to the surveillance system, and c) chikungunya and Zika cases notified to the disease surveillance system.

Although the historical time series for chikungunya and Zika incidence is short, we will nonetheless describe the incidence during and after *Wolbachia* deployments relative to the *a priori* defined non-release control areas for both Niterói and Rio de Janeiro.

### Dengue outbreak signals

As a complementary approach for evaluating the public health impact of large-scale *Wolbachia* releases, we will also use the following dengue outbreak alert tools routinely used in public health practice. We hypothesise that these dengue outbreak signals will not be triggered in areas where *Wolbachia* has been established.

**1. Control diagram (endemic channel)**

The definition of a dengue outbreak or epidemic has changed over time in Brazil. In Rio de Janeiro and Niterói, a control diagram is currently used to monitor dengue incidence. Briefly, the control diagram is constructed from a five-week centered moving average of weekly notified dengue incidence for the past five years excluding epidemic years. An early signal of a dengue outbreak/epidemic is triggered when the weekly incidence of dengue crosses the upper limit of the control diagram ^26^, with the upper limit defined as [mean+(standard deviation*1.96)]. Incidence that remains above the upper limit of the control diagram for two or more consecutive weeks constitutes a dengue outbreak. For the purpose of monitoring the impact of *Wolbachia* releases on dengue, we will construct annual control diagrams with weekly dengue incidence, by city and for each release and non-release zone, to monitor the occurrence of dengue outbreaks. The number of dengue outbreak signals triggered per year will be reported.

**2. Classical incidence threshold**

Another outbreak definition that has been used by the Ministry of Health (MoH) in previous years ^20, 22, 23^ is a dengue incidence threshold of ≥300 cases/100,000 population in a given month. Although not included in current MoH guidelines, this provides an alternative endpoint for evaluating dengue activity at a population level in the post-intervention period, compared with pre-intervention, and we will report the number of months in a given year where dengue incidence crosses this threshold.

### Current study status

This study is ongoing. *Wolbachia* releases are expected to be completed by the end of 2019, and the collation and analysis of disease surveillance data will continue until 2023.

### Dissemination of study results

Based on the results of the power estimation above, the study outcome will be evaluated and reported two years after the completion of releases. The findings will be submitted for peer review and publication in an appropriate open access journal, together with aggregate supporting data.

## Discussion

The two cities of Rio de Janeiro and Niterói in southeastern Brazil have been affected by dengue for more than 30 years, with epidemics occurring every 2 to 5 years. In recent years, outbreaks of the other *Aedes-*borne diseases chikungunya and Zika have presented further public health challenges. Vector control strategies, based on elimination of mosquito breeding sites and use of insecticides to reduce adult populations, have not been effective in preventing dengue outbreaks ^28^. The *Wolbachia* method is a novel and self-sustaining approach for the biological control of arboviral diseases. The signature feature of *Wolbachia* is to reduce the arbovirus-transmission potential of *Wolbachia-*infected mosquitoes ^12, 14, 16, 17^. The World Mosquito Program ^29^ is deploying *w*Mel *Wolbachia*-infected *Ae. aegypti* mosquitoes in Brazil with the purpose of achieving a large-scale and sustained reduction in arboviral disease burden in two cities where these diseases are public health priorities.

In March 2016, the WHO convened a Vector Control Advisory Group to review new and existing vector control tools for use in the response to the Zika virus outbreak. Based on the available evidence that *Wolbachia* reduces the Zika, dengue and chikungunya transmission potential of *Ae. aegypti* mosquitoes and field data showing long-term establishment of *Wolbachia* in mosquito populations in a range of environmental settings, the WHO recommended carefully monitored pilot implementation of the *Wolbachia* method in affected countries ^24^.

While RCTs are still considered the gold standard, they are not always feasible or agreeable to the community and government. The controlled ITS analysis is a quasi-experimental design that is commonly used to evaluate population-level public health intervention ^27, 30, 31^ and is a pragmatic alternative design where an RCT is considered infeasible, particularly in the presence of a well-matched untreated control area ^32, 33^. While the ITS approach could also be combined with randomization of the intervention, given the public health emergency posed by the Zika epidemic at the time of this study’s inception and the need to scale up *Wolbachia* deployment in Rio de Janeiro and Niterói within a relative short time frame, randomized allocation of the *Wolbachia* deployments was not considered feasible as it would have necessitated a fragmented approach to community engagement and release logistics which would have considerably complicated and delayed the implementation. The Brazil deployments were also an opportunity to optimise methods for large-scale deployment, and to evaluate the public health impact of a large contiguous release where the intervention area is likely to encompass a greater proportion of people’s daily movements, compared to a cluster randomised trial design. The controlled ITS is appropriate for the pragmatic evaluation of large-scale *Wolbachia* deployments given the long and reliable time series of dengue mandatory reporting data from both Rio de Janeiro and Niterói that allows for a longitudinal assessment of dengue trends before and after the *Wolbachia* intervention. Assessment of an impact of the intervention on chikungunya and Zika may be more difficult given their shorter time series.

Notifiable disease surveillance data can be limited by a lack of specificity in case definitions and inconsistent reporting practices, which may influence our ability to detect a true intervention effect on arboviral disease incidence. A subset of notified dengue cases are supported by laboratory diagnostic results, but these have several limitations: i) laboratory testing occurs infrequently (<15% of notified cases), particularly during outbreaks, ii) the cross-reactivity of IgM serology between dengue and Zika limits the utility of serological data since 2015, and iii) the restricted use of PCR in only certain patient populations limits the generalisability of PCR-positivity rates to all notified cases. We therefore base our analyses on all notified cases (suspected and confirmed). Benefits of using these routinely collected data include the availability of a long time series, reduced costs for data collection and timely acquisition of data. The non-randomized and unblinded nature of the *Wolbachia* intervention means it is plausible that community awareness could alter health-care seeking behaviour, diagnostic or reporting practices in areas where *Wolbachia* releases have been conducted. However we believe it is unlikely this would be of sufficient magnitude to result in a biased estimation of the intervention effect, since the intervention is deployed throughout such a large, diverse and complex setting (>120km ^2^, with population >1 million). We also cannot exclude the possibility of a concurrent change in socio-demographic or ecological factors related to dengue risk, or a change in dengue control practices, between the intervention and control areas independent of the *Wolbachia* intervention, but given the strong synchrony in dengue incidence between intervention and control zones for over a decade prior to releases this is considered unlikely. 

Human mobility also presents a potential challenge to this study, especially during the period in which staged releases are occurring and *Wolbachia* levels are heterogeneous, as individuals are likely to spend time in both *Wolbachia*-treated and untreated areas, making it difficult to determine the place of illness acquisition among notified cases of arboviral disease. Travel between areas means that the true *Wolbachia* exposure status of individuals resident in *Wolbachia*-treated and untreated areas becomes more similar, thereby diluting the observed intervention effect towards the null.

The introduction of Zika and chikungunya viruses brought new challenges to health surveillance and a greater willingness for better vector control in affected regions. With the re-emergence of yellow fever in Brazil ^8^, there is even more potential public health benefit if the *Wolbachia* intervention successfully reduces the vector competence of *Ae. aegypti* mosquitoes in the field and reduces arboviral disease incidence. No specific treatment for dengue, chikungunya or Zika currently exists. Although a vaccine against dengue (Sanofi Dengvaxia ^®^) was licensed in 2015, it was recently found to enhance the severity of subsequent dengue infection in individuals who were seronegative at the time of vaccination. As a result, a serology test prior to the administration of the vaccine is required to confirm previous dengue infection, increasing costs and decreasing feasibility in high-burden areas ^34^.

Releases of *Wolbachia* are completed or underway in eight countries, with no evidence of local transmission of dengue, Zika or chikungunya in places where *Wolbachia* is established at high levels ^21^. The implementation of the *Wolbachia* intervention is complex and has not been done on a large scale in very densely populated urban areas in the Americas before. The implementation of the project was preceded by careful work to engage the community and gain public acceptance for the intervention, even in the most difficult contexts of poverty and urban violence present in both Rio de Janeiro and Niterói. Engagement, entomological monitoring, and public health impact assessment activities were developed in close partnership with local governments. If the current project is successful, this model can be expanded to the rest of the country and the Americas.

## Ethics approval and consent to participate

The study was approved by the Brazilian Institutional Review Board (Conep) (CAAE: 59175616.2.0000.0008). The study uses pre-existing non-identifiable disease surveillance data, which does not require individuals’ consent.

## Data availability

No data is associated with this article.
