# Supplementary material for: The impact of large-scale deployment of Wolbachia mosquitoes on dengue and other Aedes-borne diseases in Rio de Janeiro and Niterói, Brazil: study protocol for a controlled interrupted time series analysis using routine disease surveillance data
Source: F1000Res. 2020 Jun 11;8:1328. Originally published 2019 Aug 1. [Version 2] doi: 10.12688/f1000research.19859.2 (PMC7780340; doi:10.12688/f1000research.19859.2)
Supplement: Peer review history

# Peer review: Reviewer response for version 2

**DOI:** 10.5256/f1000research.27081.r74998

**Published:** 2020-12-22

**Authors:** Stephen Waterman

**Affiliations:** 1 Division of Vector-Borne Diseases (DVBD), National Center for Emerging and Zoonotic Infectious Diseases (NCEZID), Centers for Disease Control and Prevention (CDC), San Juan, Puerto Rico

This manuscript describing the protocol for a intervention trial of Wolbachia replacement in *Aedes aegypti* populations with the wMel Wolbachia strain to prevent dengue, Zika and chikungunya in the complicated densely populated urban environments of Rio de Janeiro and Niteroi, Brazil, has been revised somewhat in response to previous open reviewers. In response to both reviewers, the authors explain the pragmatic reasoning behind not conducting the study as a cluster randomized trial.  In addition to community pressure to deploy the intervention quickly and in wide areas as a result of the Zika outbreak, the authors indicate that the necessary community engagement to set up clusters was not feasible given the resources available, and that the smaller geographic units are more susceptible to contamination.  In response to reviewer 1 the authors clarify the Wolbachia strain used and state that the wMel strain has persisted at high levels for over two years in Indonesia and as many 8 years in northern Australia. 

 Each city has four intervention release zones (ranging from 9 to 51 square kms and populations densities from 1,400-18,000 persons/square km) and a large non-release control area.  Adult mosquito releases took place between late 2015 and the end of 2019 and lasted for at least 16 weeks in each release zone. Wolbachia introgression frequency monitoring is done with BG traps and PCR testing of a sample of the mosquitoes collected. The study design main analysis methodology is controlled interrupted time series which makes use of routinely collected public health disease surveillance data and requires that consistent historical data is available supporting the comparability of the intervention and control areas. The analysis compares the incidence of suspect and confirmed dengue cases before and after the intervention in the treatment and control zones. Additional details on the regression analysis have been provided.   Power estimates from simulations based on binomial distribution of ten year historical data suggest that the study has 80% to detect a 50% reduction after three years, and a 60% reduction after two years. 

 This study is important to assess the impact of Wolbachia replacement in a highly Aedes aegypti transmitted disease endemic urban area in Latin America, as the other studies conducted by the World Mosquito Program have been done in Asia or in non-endemic Australia. Since the study is not randomized, including as much detail as possible in the description of the methods and the results in eventual publications will be important to assess the strengths and weaknesses of the study.

 Comments

- Additional details regarding the Wolbachia mosquito releases and trapping results would be of interest. Why were releases done as adult mosquito releases and not eggs as in the Yogyakarta Indonesia RCT trial? (1) Were releases in all release zones begun simultaneously or staggered?  Were the BG trap adult female Aedes aegypti mosquito counts/densities comparable in the different zones and control area? Will the study design enable assessment of the impact of Wolbachia releases on *Aedes* species abundance. 
- Large dengue outbreaks had not occurred prior to the intervention in the study cities since 2013. Is there available seroprevalence data for dengue and Zika in the intervention zones and the control area prior to the wMel intervention? Do we know if pre-intervention reported Zika incidence in 2016 was comparable in the intervention and control areas? 
- The analysis plan is to report results two years after completion of releases. If the releases were staggered will the initial publication include data from all the intervention zones? 
- Are the secondary endpoint analyses of severe dengue cases and fatalities intended to strengthen the efficacy evidence with data on confirmed diagnoses, to help address the cost effectiveness of the intervention, or to address a question whether Wolbachia reduces the severity of disease when transmission does occur?   
- The protocol states that the study will continue through 2023 and analyses will be done each year for 5 years. Since the releases finished at the end of 2019, will an analysis of the sustained Wolbachia introgression and cumulative epidemiologic impact after 5 years be possible?   

Minor comments

- The statement in last paragraph of page 9 that we have no evidence of dengue, Zika or chikungunya transmission where high levels of Wolbachia have been established is somewhat misleading in that the authors are referring to northern Australia where Aedes aegypti transmitted diseases are not endemic and the force of infection is considerably lower than Asia and Latin America. 
- The comment on page 9, next to last paragraph with regard to the licensed CYD-TDV vaccine being rendered less feasible in high burden areas because of the need for pre-vaccination screening actually better applies to low burden areas. WHO included in its 2018 vaccine recommendations (2) that areas with dengue seroprevalence of 80% or greater in 9 years might consider forgoing pre-vaccination screening. In low burden regions the risk of vaccinating false serologic positives increases and the cost effectiveness decreases. The authors may want to clarify whether they think Wolbachia replacement could make dengue vaccines unnecessary. 

Is the study design appropriate for the research question?

Yes

Is the rationale for, and objectives of, the study clearly described?

Yes

Are sufficient details of the methods provided to allow replication by others?

Partly

Are the datasets clearly presented in a useable and accessible format?

Not applicable

Reviewer Expertise:

Epidemiology, dengue and arbovirology, tropical medicine

I confirm that I have read this submission and believe that I have an appropriate level of expertise to confirm that it is of an acceptable scientific standard.

## References

[1] Weekly Epidemiological Record Dengue Vaccine. *WHO*.36(93) :457-476 Reference source

# Peer review: Reviewer response for version 1

**DOI:** 10.5256/f1000research.21787.r57000

**Published:** 2019-12-09

**Authors:** Adeshina I. Adekunle

**Affiliations:** 1 Australian Institute of Tropical Health and Medicine, James Cook University, Townsville, Australia

**Review:**

 The authors present the study protocol for an ongoing large deployment of *Wolbachia-*infected *Aedes-aegypti* mosquitoes in Rio de Janeiro and Niteroi municipalities in southeastern Brazil where *Aedes-*borne diseases, especially dengue, are common. The study design is a quasi-experimental study (QES) that allows the application of the controlled interrupted time series analysis to evaluate whether *Wolbachia* strategy is effective in this region. The manuscript is well written and the study, when completed, has great potential for evaluating the promising *Wolbachia *strategy for controlling *Aedes*-borne diseases. However, I have some concerns about this study and the ability of the interrupted time series method to infer reduction in disease burden if present:

**Main comments:**

- *Aedes-aegypti* mosquitoes movement.

First of all, the authors failed to indicate the strain of *Wolbachia* used in this study. Different strains of *Wolbachia *have different characteristics that enable them to invade the wild type population and establish themselves. The *wMel* strain was used in Townsville, Australia ^1^ and was claimed to be successful. However, with climate change and variability in temperature, some *Wolbachia-*infected mosquitoes with different strains, include *wMel, *cannot maintain their *Wolbachia *status ^2^. Hence, even if the intervention works, an introduction of infected patients into such a population will result in an outbreak and all the gains from *Wolbachia *strategy would have been lost as the majority of the mosquitoes are now uninfected ^3^.

 Figure 1 in the manuscript shows the map of the treated and untreated zones. These are adjacent zones, especially for the treated and untreated zones. Mosquitoes movement from untreated zone to treated zone could prevent *Wolbachia *invasion ^4^ and vice versa. Hence, and since this is a study that takes a few years, mosquitoes migration cannot be ruled out. Thus, the control zones (untreated) is not suitable anymore.

- The controlled interrupted time-series method.

The authors stated that the reason for favoring quasi-experimental design (QED) over randomized controlled trial (RCT) was due to time constraints as stakeholders and funders wanted to scale-up the deployment of *Wolbachia *to accelerate the control of the emergent Zika virus in this region. However, in this set-up, to carefully randomize zones as treated or not should not take much time. In fact, since the incidence of *Aedes-borne* virus infections is similar between treated and untreated zones (Figure 2), that should be a motivation for randomizing the zones. I am thinking there are other reasons for ditching RCT. The authors should elaborate on this.

 For the controlled interrupted analysis, the authors plan to perform first, the descriptive analysis of the seasonal and inter-annual trends in the *Aedes-borne* infection notifications. It will be great if the authors are more specific about the types of descriptive analysis they intend to carry out. Also, a schematic of the expected trend in the notifications accounting for confounders will greatly help the protocol.

**Minor comments:**

**Title**

1. The title indicated controlling “arboviral disease incidence”. The authors can be more specific if they change that to either “ *Aedes*-borne disease incidence” or something more specific.

**Abstract**

1. I think the clause in the fourth line will flow better if “ *Aedes*-borne diseases dengue, chikungunya and Zika” is changed to  “ *Aedes*-borne diseases (dengue, chikungunya and Zika).
2. Change “km2” to “km ^2^”.

**Methods**

1. Figure 2 shows the comparative incidence of dengue for the treated and untreated regions. What about Zika and Chikungunya that the study is trying to control too? I think this is a dengue control study that has the potential to control Zika and Chikungunya in these regions. It will be great if the authors make it appear so. As it is, it seems to be a study deliberately targeting all the three *Aedes-*borne diseases.

Is the study design appropriate for the research question?

Yes

Is the rationale for, and objectives of, the study clearly described?

Yes

Are sufficient details of the methods provided to allow replication by others?

Yes

Are the datasets clearly presented in a useable and accessible format?

Not applicable

Reviewer Expertise:

Infectious disease modelling and bio-statistics

I confirm that I have read this submission and believe that I have an appropriate level of expertise to confirm that it is of an acceptable scientific standard.

## References

[1] : Scaled deployment of Wolbachia to protect the community from dengue and other Aedes transmitted arboviruses. *Gates Open Res*.2018;2: 10.12688/gatesopenres.12844.3 36 10.12688/gatesopenres.12844.3 30596205PMC6305154

[2] : The Wolbachia strain wAu provides highly efficient virus transmission blocking in Aedes aegypti. *PLoS Pathog*.14(1) : 10.1371/journal.ppat.1006815 e1006815 10.1371/journal.ppat.1006815 29370307PMC5784998

[3] : Mathematical analysis of a Wolbachia invasive model with imperfect maternal transmission and loss of Wolbachia infection. *Infect Dis Model*.2019;4: 10.1016/j.idm.2019.10.001 265-285 10.1016/j.idm.2019.10.001 31709323PMC6829679

[4] : Modelling the spread of Wolbachia in spatially heterogeneous environments. *J R Soc Interface*.2012;9(76) : 10.1098/rsif.2012.0253 3045-54 10.1098/rsif.2012.0253 22675165PMC3479901

# Response

**Published:** 2020-06-01

**Authors:** Katherine ANDERS

**Affiliations:** Monash University, Australia

**Major comments**

****

**1.    The authors failed to indicate the strain of Wolbachia used in this study. Different strains of Wolbachia have different characteristics that enable them to invade the wild type population and establish themselves. The wMel strain was used in Townsville, Australia and was claimed to be successful. However, with climate change and variability in temperature, some Wolbachia-infected mosquitoes with different strains, include wMel, cannot maintain their Wolbachia status. Hence, even if the intervention works, an introduction of infected patients into such a population will result in an outbreak and all the gains from Wolbachia strategy would have been lost as the majority of the mosquitoes are now uninfected.**

 Thank you for drawing our attention to this oversight. We have now indicated the strain used (wMel) in the abstract, the sixth paragraph of the introduction, the ‘Wolbachia release and monitoring’ subheading of the Methods, and the first paragraph of the discussion. While it is true that other Wolbachia strains have been shown to have higher thermostability than wMel in the mosquito larval life stages, the effect of heatwaves on wMel Wolbachia prevalence in Wolbachia-treated areas of northern Australia has been shown to be transient (Ross et al, PLoS NTD 2020). Long-term monitoring of WMP’s release sites in northern Australia and Yogyakarta, Indonesia, has demonstrated sustained high levels of Wolbachia 2 – 8 years post-release. Entomological monitoring of Wolbachia prevalence in local Ae. aegypti populations in the Rio and Niteroi release areas is currently ongoing, and so will provide empirical evidence of the level to which wMel introgression is sustained over time.

**2.    Figure 1 in the manuscript shows the map of the treated and untreated zones. These are adjacent zones, especially for the treated and untreated zones. Mosquitoes movement from untreated zone to treated zone could prevent Wolbachia invasion4 and vice versa. Hence, and since this is a study that takes a few years, mosquitoes migration cannot be ruled out. Thus, the control zones (untreated) is not suitable anymore.**

 It is true that Wolbachia could theoretically spread from the deployment areas into the untreated control zones, however the rate of spread is known to be relatively slow especially in dengue urban environments like these (100-200m per year; Schmidt et al PLoS Biol 2017). Given the size of the control zone (117km2 in Rio de Janeiro, and 51km2 in Niteroi), it is highly unlikely that Wolbachia establishment will occur in any meaningful way beyond the border area of the control zones within the 5 year timeframe of this study. 

**3.    The authors stated that the reason for favoring quasi-experimental design (QED) over randomized controlled trial (RCT) was due to time constraints as stakeholders and funders wanted to scale-up the deployment of Wolbachia to accelerate the control of the emergent Zika virus in this region. However, in this set-up, to carefully randomize zones as treated or not should not take much time. In fact, since the incidence of Aedes-borne virus infections is similar between treated and untreated zones (Figure 2), that should be a motivation for randomizing the zones. I am thinking there are other reasons for ditching RCT. The authors should elaborate on this.**

 The randomization itself would not necessarily take a lot of time, although there would be additional work required to define an adequate number of spatial units for randomization (ideally based on a power calculation), and define those spatial units with ‘hard’ borders which minimise contamination between treated and untreated areas. The time delays come from the added complexity of conducting the intensive community and stakeholder engagement that is needed prior to releases, and conducting the deployments themselves, across a fragmented set of release areas interspersed with untreated control areas, compared to conducting these engagement and deployment activities in a contiguous release area. Please see our response to Reviewer 1’s comments #1, #7 and #11 for further discussion of the reasons for non-randomisation, and for the references to where we have improved discussion of this point in the manuscript text.

**4.    For the controlled interrupted analysis, the authors plan to perform first, the descriptive analysis of the seasonal and inter-annual trends in the Aedes-borne infection notifications. It will be great if the authors are more specific about the types of descriptive analysis they intend to carry out. Also, a schematic of the expected trend in the notifications accounting for confounders will greatly help the protocol**

 The descriptive analysis will simply describe the seasonal and interannual trends, as stated. I.e. the calendar months in which dengue incidence has historically been highest and lowest in the two cities, and the range in annual case counts and per capita incidence. We don’t see that this level of detail is of value to the reader here. We don’t understand the reviewer’s suggestion about a schematic of expected trend in notifications. We show in figure 2 the historical trends in dengue incidence in the intervention area and untreated control area for each city; the lines for intervention and control area can be expected to diverge if Wolbachia successfully interrupts dengue transmission.

 Minor comments

**5.    The title indicated controlling “arboviral disease incidence”. The authors can be more specific if they change that to either “Aedes-borne disease incidence” or something more specific.**

 We have changed the wording in the title to ‘Aedes-borne disease’ as suggested.

**6.    I think the clause in the fourth line [of the abstract] will flow better if “Aedes-borne diseases dengue, chikungunya and Zika” is changed to  “Aedes-borne diseases (dengue, chikungunya and Zika).**

 We have kept the original wording here, because strictly these three are not the only Aedes¬-borne diseases, but they are the Aedes-borne diseases were are considering in this protocol.

**7.    Change “km2” to “km2”**

 This change has been made.

**8.    Figure 2 shows the comparative incidence of dengue for the treated and untreated regions. What about Zika and Chikungunya that the study is trying to control too? I think this is a dengue control study that has the potential to control Zika and Chikungunya in these regions. It will be great if the authors make it appear so. As it is, it seems to be a study deliberately targeting all the three Aedes-borne diseases**

 See response to comment #13, Reviewer 1.

 Historical chikungunya data for the intervention areas and control areas is now included as Figure 3.

# Peer review: Reviewer response for version 1

**DOI:** 10.5256/f1000research.21787.r51960

**Published:** 2019-11-07

**Authors:** Liz Turner

**Affiliations:** 1 Department of Biostatistics and Bioinformatics, Duke University, Durham, NC, USA

This very well written manuscript describes the protocol for a quasi-experimental study using controlled interrupted time series. The study is currently being conducted in Brazil. The selected design is an innovative use of the controlled interrupted time series which can leverage routinely collected surveillance data to seek to address causal questions of interest. Moreover, the intervention being evaluated, namely the deployment of *Wolbachia* mosquitoes with the goal to reduce arboviral disease incidence, is innovative and shows great promise.

 I have two main comments:

- A desire to see more justification for why randomization was not possible. The difficulties of randomization in this setting are mostly described but, as you’ll see from my more detailed comments below, it’s not absolutely convincing that randomization would not have been possible here, even within the interrupted time series design.
- Because the measurement of incidence of the arbobviruses of interest is mostly through surveillance data and it may be difficult to blind communities to the use of *Wolbachia*, how can it be determined that in the treatment zones post- *Wolbachia* release any smaller incidence is not just related to the fact that there is lower reporting as community members expect the incidence of those viruses to go down? I have a big concern about the possibility of differential assessment of outcomes in treated and control zones. Can the authors elaborate on this in relevant places in the text?

Here I offer some more minor points that may help with clarity for the reader:

**Abstract**

- It could help the reader if when “dengue, chikungunya and Zika” are first mentioned in the abstract, they are also named as arboviruses i.e. to help readers not necessarily familiar with them to be certain that all three fall in to this class of viruses.

**Background**

- Based on the title referring to arboviruses, as a reader, I was expecting the first sentence or so to at least mention of arbovirus. Would it be possible to do so? This could be achieved very easily by saying something like “ *The global incidence of the arbovirus dengue has increased dramatically in recent decades*.”
- In the 4 ^th^ paragraph referring to yellow fever, please clarify if is an arbovirus.
- The last sentence of the 6 ^th^ paragraph: “Laboratory data indicate a similar reduction in the competence of *Wolbachia*-carrying Ae. aegypti for transmitting other viruses including Zika, chikungunya, yellow fever and Mayaro”. If all listed are arboviruses, please edit “viruses” to “arboviruses”.
- In the 9 ^th^ paragraph, I very much appreciate the rationale that there was demand for scale-up of implementation of the *Wolbachia* releases. I would appreciate a little more clarity on why randomization was not possible since, given that not all areas of the study will receive the treatment during the study, the scale-up argument doesn’t seem sufficient to justify no randomization. I can very well imagine that there are many compelling reasons to not randomize, some of which are alluded to, and so it would help to be even clearer about this e.g. was it because of the existing partnerships and infrastructure making certain areas better for release than others? What else? Some of this is sort of implied in the text and it would be valuable to have just a few more explicit sentences regarding this.
- Related to my previous comments on arboviruses, in the 10 ^th^ paragraph where it is stated: “Here we describe a protocol for evaluating the effect of large scale non-randomized *Wolbachia* releases on the incidence of dengue, Zika and chikungunya in the municipalities of Niterói and Rio de Janeiro.”, again, it could really help the reader to edit this sentence to something like “…on the incidence of three arboviruses, namely dengue, Zika and chikungunya, in the……”.
- Also, see a later comment based on the discussion as to why yellow fever is not also included in the study.

**Methods – Study design**

- Why do the two stated objectives only relate to dengue? It will be important to make clear in the manuscript as it is strange that the overall goals are related to three arboviruses but the objectives only related to one of them.
- Table 1: Are zones 1-3 in Niteroi and zones 1-3.2 in Rio the release areas? If so, please more clearly label this in the table as it is not immediately obvious to me when I look at the table even though I infer that it is the case. Perhaps add a sub-heading of “release areas” above the rows where the names of the release zones are provided.

**Methods – Study setting and population** 

- Overall, the rationale appears reasonable for the use of controlled interrupted time series but, in reference to my comments above, please look for some opportunities to be even clearer about why randomization could not be used. An interrupted time series design could still be adopted, but one with randomization could offer even stronger evidence.
- “In Rio de Janeiro, two administrative areas adjacent to the release area have been designated a priori as a comparative control zone (Figure 1), based on comparable sociodemographic characteristics and synchronous historical dengue time series (Figure 2 and Table 1).” I do not see any information on these SES characteristics except for population whereas I see the “synchronous historical dengue time series” data in Figure 2, which is really helpful. Can more info on SES be added to the appendix or is the data presented the extent of the available data? As a reader, I would want that information when judging the validity of the comparisons between treatment and control areas. Incidentally, is no historical data available on Zika and Chikungunga? If it is available, it would be really valuable to see that compared between treated and control. I see in the last paragraph of pg 6 that some such data is available so why not include it in the current manuscript?

**Controlled interrupted time series**

- It is stated: “Zone-level ITS analyses will be performed 12 months after completion of releases in each zone, and each 12 months thereafter, with release zones considered ‘treated’ for the purpose of this analysis based on completion of releases, regardless of the long-term *Wolbachia* monitoring results.” What does “performed 12 months after completion”? Is it literally about the timing of running the analyses, even though data from each month will be used? The reason I ask for clarification is that on first read I thought that this meant there was a switch to only using data at 12 months after completion of the releases. Also, what is the time frame for control areas where there is no release? Please be more specific.
- “Aggregate release area level analyses will be performed for each municipality 12 months after completion of releases in the last zone and each 12 months thereafter.” What are municipalities? Please define this term when it is first introduced as I don’t see this term defined elsewhere. Is it city i.e. Rio and Niteroi? If so, why not just use the same term throughout i.e. municipality or city, and only choose one of those terms?
- Regarding analyses, I recognize that there may not be space but it would be beneficial to provide some more details including:
   
  - Model diagnostics to be used e.g. what happens if there is even more dispersion than can be accommodated than the proposed negative binomial models?
  - How will contrasts be made between the treated and control communities? As far as I can tell, this is not specified, though perhaps I’m missing it? Please specific which parameters will be used to estimate the “treatment” effect.
  - How will the fact that the control zones are not randomized be accounted for in modelling i.e. although the set of control zones were selected to be comparable on average to those treated based on historical dengue incidence and SES, what about other factors that may be different between the treated and control zones?
  - Will there be a “fake” interruption in the series for the control areas? It doesn’t seem necessary and may be problematic but would be valuable to describe explicitly what will be done in the time series modelling for the control zones.

**Discussion**

- The discussion starts with a sentence on dengue, zika and yellow fever. Why is yellow fever not included in the outcome measures for the proposed study?

Overall, this is a fascinating study and a great use of an interrupted time series.

Is the study design appropriate for the research question?

Yes

Is the rationale for, and objectives of, the study clearly described?

Yes

Are sufficient details of the methods provided to allow replication by others?

Partly

Are the datasets clearly presented in a useable and accessible format?

Not applicable

Reviewer Expertise:

Statistical methods in global health research including elimination science and impact evaluation with a focus on malaria and febrile illnesses

I confirm that I have read this submission and believe that I have an appropriate level of expertise to confirm that it is of an acceptable scientific standard, however I have significant reservations, as outlined above.

# Response

**Published:** 2020-06-01

**Authors:** Katherine ANDERS

**Affiliations:** Monash University, Australia

**Main comments**

**1.    I have a desire to see more justification for why randomisation was not possible. The difficulties of randomisation in this setting are mostly described but, as you’ll see from my more detailed comments below, it’s not absolutely convincing that randomisation would not have been possible here, even within the interrupted time series design.**

 We agree with the reviewer that randomisation could be combined with ITS, and in principle this could be a stronger design. However randomisation of the deployment (either by random allocation of parallel treated and untreated areas, or by randomising the sequence of staged deployments) would have necessitated the study site being divided up into a large number of spatial units for randomisation. Logistically, this would have made the community engagement and deployment activities infeasible within the funding and time constraints of the project, and in the context of the large and complex urban setting. Intensive stakeholder and community engagement, and deployment planning, could only have commenced after the randomisation process had occurred and the intervention areas were determined. Field activities would then have had to be conducted in a spatially fragmented way, complicating the stakeholder and community engagement and the deployment logistics and inevitably lengthening time frames. Smaller non-contiguous release areas interspersed with untreated areas would also have greatly increased the potential for contamination between treatment arms from both mosquito movement and human movement, thereby diluting the observable intervention effect. Please see our responses to comments #7 and #11 below, and Reviewer 2 comment #3.

**2.    Because the measurement of incidence of the arboviruses of interest is mostly through surveillance data and it may be difficult to blind communities to the use of Wolbachia, how can it be determined that in the treatment zones post-Wolbachia release any smaller incidence is not just related to the fact that there is lower reporting as community members expect the incidence of those viruses to go down? I have a big concern about the possibility of differential assessment of outcomes in treated and control zones. Can the authors elaborate on this in relevant places in the text?**

 It is plausible that community awareness could alter health-care seeking behaviour, or diagnostic / reporting practices, in areas where Wolbachia releases have been conducted. However we find it unlikely that this could lead to a large and sustained difference in dengue case notifications across two cities as large and complex as these, where the total intervention area exceeds 120km2 and 1 million inhabitants. Dengue (and chikungunya and Zika) is a notifiable disease, so there remains a requirement for health care providers to report any suspected cases meeting the standard case definition. We have included a discussion of this as potential limitation, in the fourth paragraph of the discussion. From the beginning of the project's engagement with communities, all messaging has reinforced the continued importance of routine activities related to arbovirus surveillance and care. 

**Minor comments**

****

**3.    It could help the reader if when ‘dengue, chikungunya and Zika’ are first mentioned in the abstract, they are also named as arboviruses i.e. to help readers not necessarily familiar with them to be certain that all three fall in to this class of viruses.**

 Since the three arboviruses of interest are specifically those vectored by Aedes mosquitoes, for greater clarity we have replaced ‘arboviruses’ with ‘Aedes-borne viruses’ in most instances throughout the abstract and article text, including in the manuscript title. Elsewhere we have replaced ‘arboviral disease’ with the more intuitive term ‘mosquito-borne disease’. In the last sentence of paragraph one, it is made clear that the term ‘arbovirus’ encompasses dengue, chikungunya, Zika, yellow fever and Mayaro viruses, and that these are specifically Aedes-borne arboviruses.

**4.    Based on the title referring to arboviruses, as a reader I was expecting the first sentence or so to at least mention arbovirus. Would it be possible to do so? This could be achieved very easily by saying something like “The global incidence of the arbovirus dengue has increased dramatically in recent decades”.**

 As above, the manuscript title has been adjusted to replace ‘arboviral disease’ with ‘Aedes-borne disease’ to improve clarity for the reader. The first sentence introduces dengue (the disease) as a global public health challenge, and the last sentence of that paragraph defines dengue, chikungunya and Zika (and yellow fever and Mayaro) as Aedes-borne diseases (and arboviruses). A definition of arbovirus as meaning a mosquito-borne virus has also been added to this sentence.

**5.    In the fourth paragraph referring to yellow fever, please clarify if it is an arbovirus.**

 Yellow fever has been defined as an Aedes-borne arboviruses in the last sentence of the first paragraph of the introduction.

**6.    The last sentence of the sixth paragraph: “Laboratory data indicate a similar reduction in the competence of Wolbachia-carrying Ae. aegypti for transmitting other viruses include ZIka, chikungunya, yellow fever and Mayaro”. If all listed are arboviruses, please edit ‘viruses’ to ‘arboviruses’.**

 The text has been corrected as suggested.

**7.    In the ninth paragraph, I very much appreciate the rationale that there was demand for scale-up of implementation of the Wolbachia releases. I would appreciate a little more clarity on why randomization was not possible since, given that not all areas of the study will receive the treatment during the study, the scale-up argument doesn’t seem sufficient to justify no randomization. I can very well imagine that there are many compelling reasons to not randomize, some of which are alluded to, and so it would help to be even clearer about this e.g. was it because of the existing partnerships and infrastructure making certain areas better for release than others? What else? Some of this is sort of implied in the text and it would be valuable to have just a few more explicit sentences regarding this.**

 Please see our response to Major comment #1 above. We have added text to the end of the ninth paragraph of the introduction, expanding on this justification for not randomising, as suggested.

**8.    Related to my previous comments on arboviruses, in the tenth paragraph…again it could really help the reader to edit this sentence to something like “…three arboviruses, namely dengue, Zika and chikungunya…”**

 Consistent with our response to comment # above, we have adjusted the wording in the paragraph to refer to dengue, Zika and chikungunya as ‘Aedes-borne diseases’ or ‘mosquito-borne diseases’.

**9.    Why do the two stated objectives only relate to dengue? It will be important to make clear in the manuscript as it is strange that the overall goals are related to three arboviruses but the objectives only related to one of them.**

 Thank you for pointing out this inconsistency, we have corrected the wording of the aims and first objective to be inclusive of dengue, chikungunya and Zika (as the Aedes-borne diseases of interest). The second objective still relates only to dengue, as the outbreak indicators used routinely for public health purposes in Rio and Niteroi apply only to dengue outbreak monitoring. As explained under ‘Controlled interrupted time series analysis’ (3rd and 4th paragraphs, page 11), the primary endpoint for the ITS analysis will be dengue for which there is the longest historical case time series, with chikungunya and Zika analysed as secondary endpoints in the ITS.

**10.     Table 1: Are zones 1-3 in Niteroi and zones 1-3.2 in Rio the release areas? If so, please more clearly label this in the table as it is not immediately obvious to me. Perhaps add a sub-heading of “release areas” above the rows where the names of the release zones are provided.**

 Thank you for this suggestion, we have improved the labelling of the areas in Table 1 to make clearer which are the release zones and non-release areas. Please note that we have also updated this table and Figure 2 to reflect an additional release zone in Niteroi (and the consequent reduced size of the control area).

**11.    Overall the rationale appears reasonable for the use of controlled interrupted time series but, in reference to my comments above, please look for some opportunities to be even clearer about why randomisation could not be used. An interrupted time series design could still be adopted, but one with randomisation could offer even stronger evidence.**

 Please see our responses to comments #1 and #7 above. Additional discussion of the justification for not randomising, and the potential benefits from the pragmatic evaluation of a large-scale contiguous release, has been added to the third paragraph of the discussion section on page 12.

**12.    “In Rio de Janeiro, two administrative areas adjacent to the release area have been designated a priori as a comparative control zone (Figure 1), based on comparable sociodemographic characteristics and synchronous historical dengue time series (Figure 2 and Table 1).” I do not see any information on these SES characteristics except for population whereas I see the “synchronous historical dengue time series” data in Figure 2, which is really helpful. Can more info on SES be added to the appendix or is the data presented the extent of the available data? As a reader, I would want that information when judging the validity of the comparisons between treatment and control areas.**

 Unfortunately socio-economic data are not available aggregate to the release zones. We have now corrected this text to clarify that the comparability of the untreated control areas is demonstrated by the synchronous historical dengue time series.

**13.    Incidentally, is no historical data available on Zika and Chikungunga? If it is available, it would be really valuable to see that compared between treated and control. I see in the last paragraph of pg 6 that some such data is available so why not include it in the current manuscript?**

 Historical chikungunya data for the intervention areas and control areas is now included as Figure 3.

**14.    It is stated: “Zone-level ITS analyses will be performed 12 months after completion of releases in each zone, and each 12 months thereafter, with release zones considered ‘treated’ for the purpose of this analysis based on completion of releases, regardless of the long-term Wolbachia monitoring results.” What does “performed 12 months after completion”? Is it literally about the timing of running the analyses, even though data from each month will be used? The reason I ask for clarification is that on first read I thought that this meant there was a switch to only using data at 12 months after completion of the releases. Also, what is the time frame for control areas where there is no release? Please be more specific.**

 Yes, this is about the timing of running the analyses, ie. the intervention effect will be estimate for each zone when 12 months of post-release observations have accumulated in that zone, and repeated each 12 months thereafter (for five years) on cumulative post-release observations. And the same for the city-level analyses. The text at the end of the first paragraph under the heading ‘Controlled interrupted time series analysis’ has been edited to clarify this. There is no separate analysis for control areas, these are included in the regression model as the comparator for each individual release zone, and for the city-level analysis. This has been clarified in the text.

**15.    “Aggregate release area level analyses will be performed for each municipality 12 months after completion of releases in the last zone and each 12 months thereafter.” What are municipalities? Please define this term when it is first introduced as I don’t see this term defined elsewhere. Is it city i.e. Rio and Niteroi? If so, why not just use the same term throughout i.e. municipality or city, and only choose one of those terms?**

 We recognise this inconsistency may have been confusing, and have changed throughout to use ‘city/cities’ in place of ‘municipality/municipalities’.

**16.    Model diagnostics to be used e.g. what happens if there is even more dispersion than can be accommodated than the proposed negative binomial models?**

 Of the common count models, negative binomial models have the most flexible approach for handling overdispersion in count data by assuming that the variance is a quadratic function of the mean. Common causes of overdispersion can range from an excess of zero counts, unexplained heterogeneity, and/or temporal autocorrelation. The models as proposed will be using flexible cubic splines to explain secular and temporal trends, hopefully leaving very little remaining unexplained heterogeneity. To account for any lingering heteroskedasticity or temporal autocorrelation, heteroskedasticity-robust estimation will be used for estimating variance. This has now been made explicit in the description of the Controlled interrupted time series methods. If an excess of zero counts is apparent, the proposed negative binomial model will be nested within a zero-inflated negative binomial model of the same structure. A likelihood ratio test can be performed to test model fit. This is also now described in the methods.

**17.    How will contrasts be made between the treated and control communities? As far as I can tell, this is not specified, though perhaps I’m missing it? Please specific which parameters will be used to estimate the “treatment” effect**

 The regression model will use monthly dengue case time series data for the treated area (either an individual release zone or aggregate city-level release area, as specified in the text) and the control area (one control area per city). The treatment effect will be estimated from the interaction between a binary ‘area’ variable (treated vs control) and a binary ‘period’ variable (post-Wolbachia vs pre-Wolbachia). This allow explicitly for a level change in the outcome (dengue case incidence) in both intervention and control arms in the post-intervention period, for example in a scenario where other secular events coincident with the Wolbachia deployments may have influenced dengue incidence in both intervention and control areas independent of Wolbachia. The text under ‘Controlled interrupted time series’ has been revised to clarify this.

**18.    How will the fact that the control zones are not randomized be accounted for in modelling i.e. although the set of control zones were selected to be comparable on average to those treated based on historical dengue incidence and SES, what about other factors that may be different between the treated and control zones?**

 Quasi-experimental designs, such as the proposed ITS analysis, aim to address the validity concerns associated with non-randomization in several ways. By using a region's historic data to inform the counterfactual outcome, the ITS analysis aims to minimize threats to internal validity. Second, by adding control regions and performing a controlled ITS, we better account for seasonal and secular influences during both the pre- and post-intervention periods. While it is true that there may be other factors associated with dengue risk that differ between treated and control zones in a non-randomised design, we make the implicit assumption in the controlled ITS that these differences are consistent throughout both the pre- and post-intervention period. Any divergence in the post-intervention period from the synchrony in dengue time series observed between intervention and control zones in the pre-intervention period is therefore assumed to represent an intervention effect. However we cannot exclude the possibility of a concurrent change in socio-demographic or ecological factors related to dengue risk, or a concurrent change in dengue control practices, between the intervention and control areas independe of the Wolbachia intervention. We have expanded on this limitation in the discussion.

**19.    Will there be a “fake” interruption in the series for the control areas? It doesn’t seem necessary and may be problematic but would be valuable to describe explicitly what will be done in the time series modelling for the control zones.**

 The inclusion of the control area time series in the regression model and estimation of the intervention effect from an interaction between ‘area’ and ‘period’ effectively does allow for a ‘fake’ interruption in the series for the control area, as it allows for a step change in the control area as well as the treated area, post-intervention. The interaction parameter from which the intervention effect is estimated indicates whether there was any change in dengue incidence following the Wolbachia intervention in areas where Wolbachia releases occurred, over and above any change in dengue incidence that was observed in the untreated control area.

**20.    The discussion starts with a sentence on dengue, zika and yellow fever. Why is yellow fever not included in the outcome measures for the proposed study?**

 Yellow fever is not included as an endpoint for the study because there is not a history of urban yellow fever in the cities of Rio and Niteroi. The recent outbreak in Rio de Janeiro state that is mentioned early in the discussion included some urban cases, but in other parts of the state. We have removed that sentence from the first paragraph of the discussion, to avoid confusion.
